# Supplementary material for: Non-invasive prenatal testing (NIPT) by low coverage genomic sequencing: Detection limits of screened chromosomal microdeletions
Source: PLoS One. 2020 Aug 26;15(8):e0238245. doi: 10.1371/journal.pone.0238245 (PMC7449492; doi:10.1371/journal.pone.0238245)
Supplement: S1 Text — (PDF) [file pone.0238245.s001.pdf]

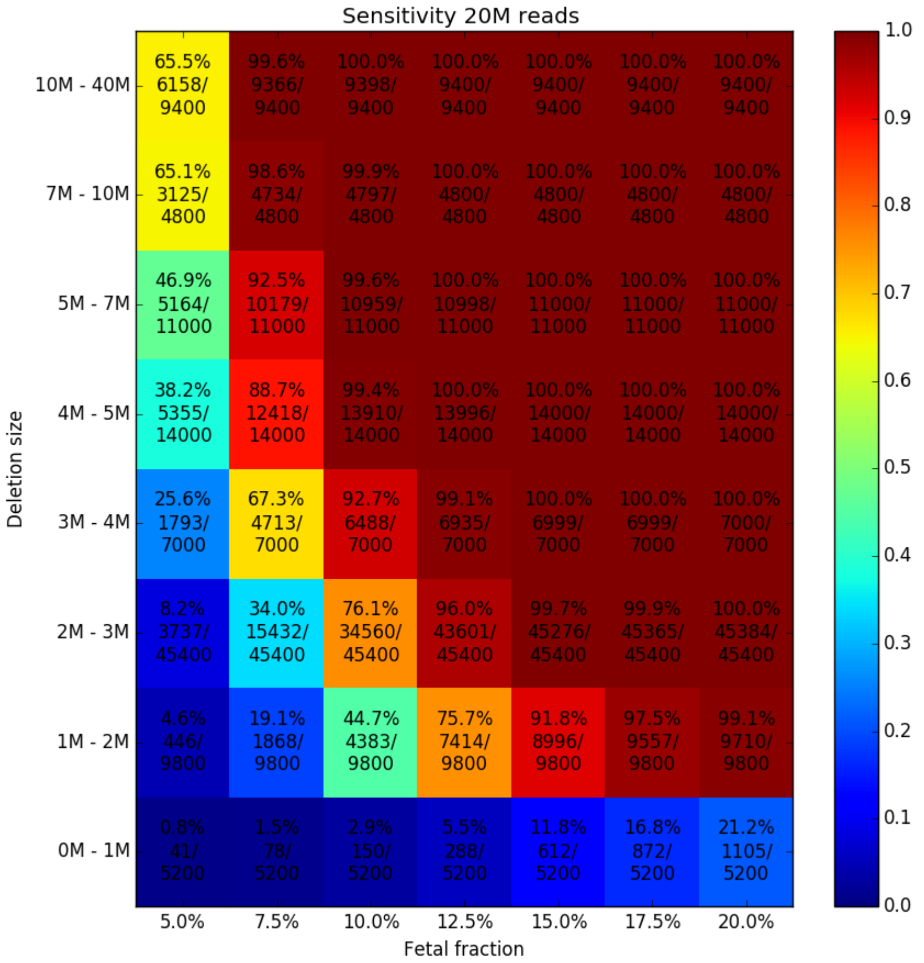

Fig A. Sensitivity of the prediction for different fetal fraction and microdeletion size.
Read count was set to 20M in each sample. Detections 2Mb away from critical region are reported.

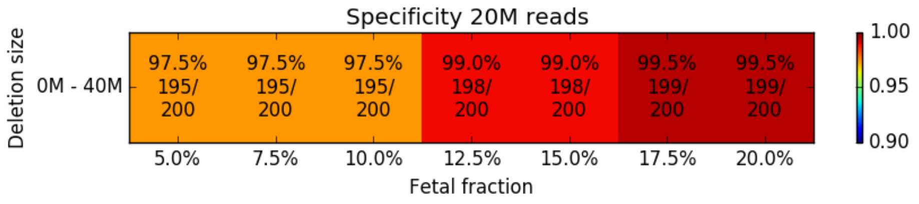

Fig B. Specificity of the prediction for different fetal fraction and microdeletion size.
Read count was set to 20M in each sample. Detections 2Mb away from critical region are reported.

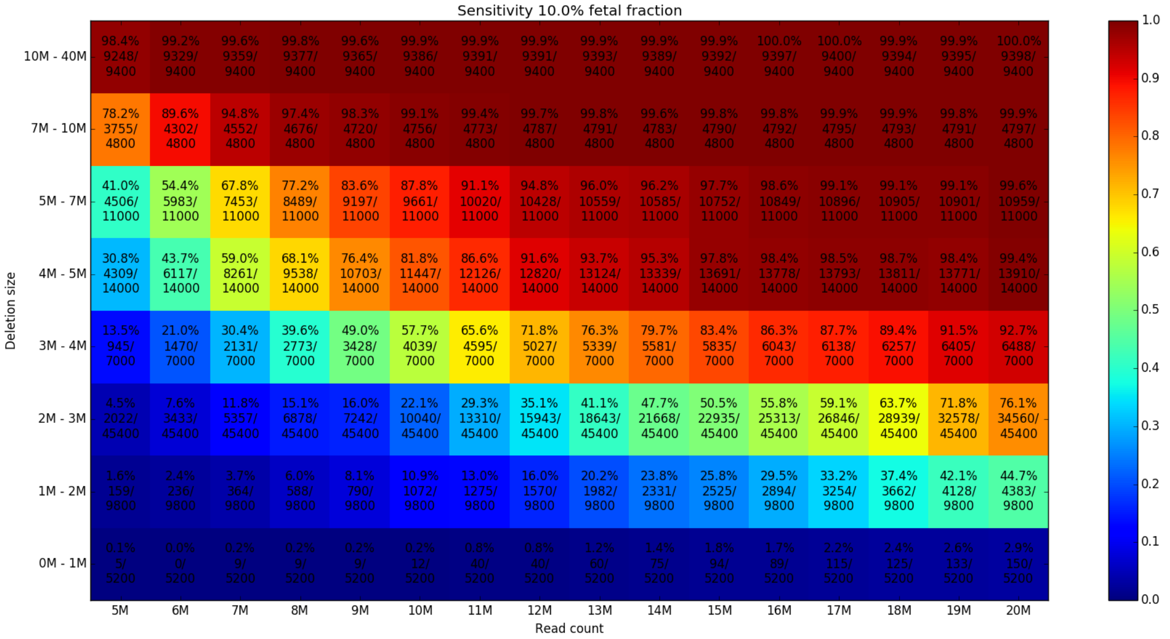

Fig C. Sensitivity of the prediction for different read count and microdeletion size.

Fetal fraction was set to 10% for all samples. Detections 2Mb away from critical region are reported.

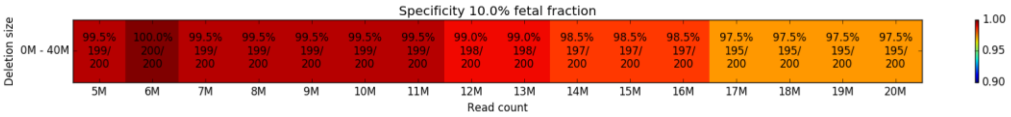

Fig D. Specificity of the prediction for different read count and microdeletion size.

Fetal fraction was set to 10% for all samples. Detections 2Mb away from critical region are reported.

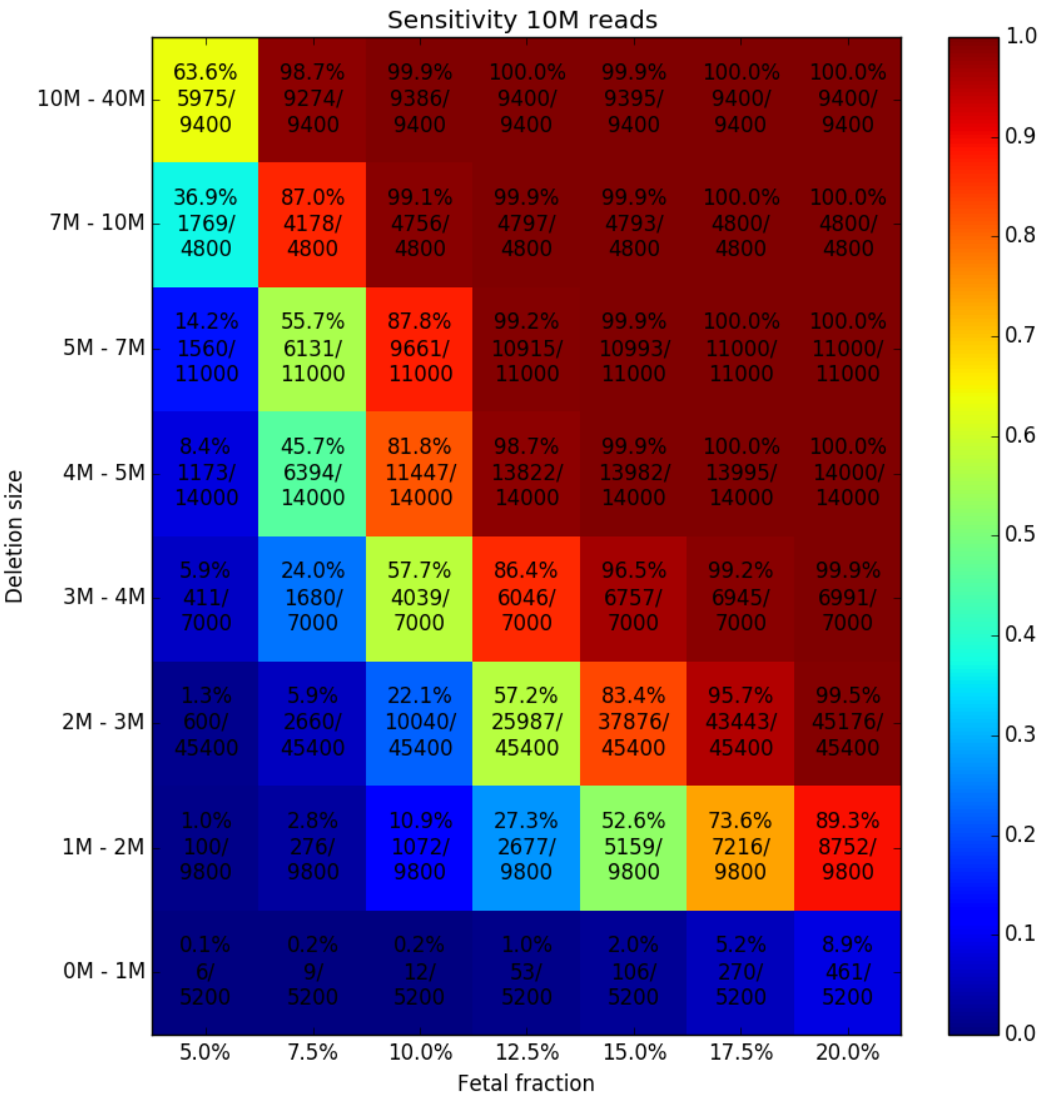

Fig E. Sensitivity of the prediction for different fetal fraction and microdeletion size.

Read count was set to 10M in each sample. Detections 2Mb away from critical region are reported.

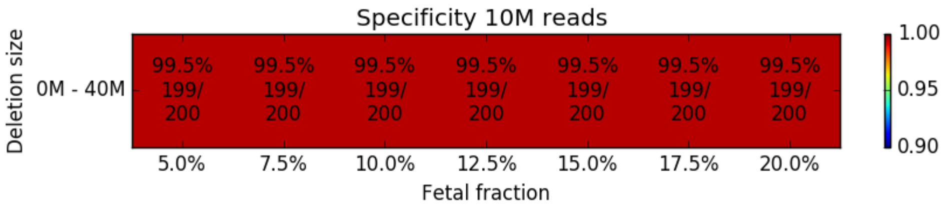

Fig F. Specificity of the prediction for different fetal fraction and microdeletion size.

Read count was set to 10M in each sample. Detections 2Mb away from critical region are reported.

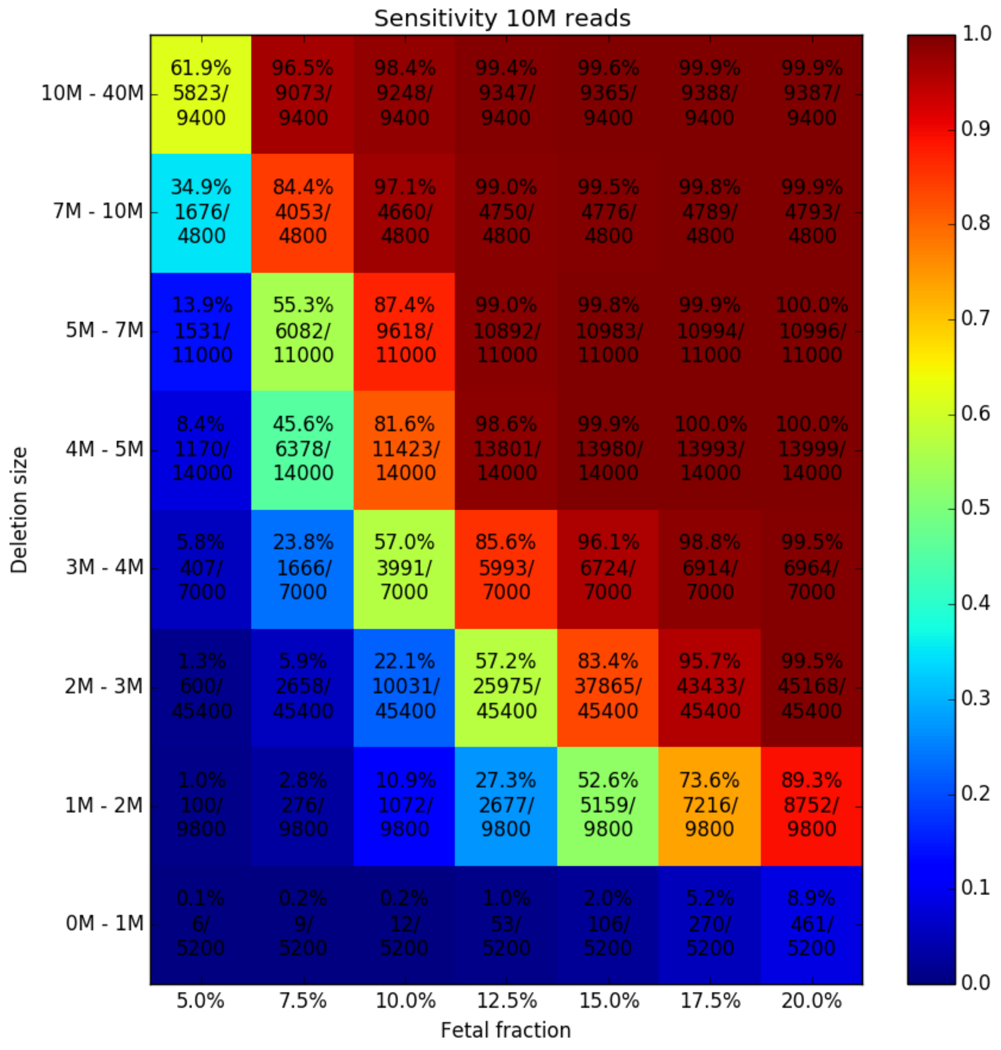

Fig G. Sensitivity of the prediction for different fetal fraction and microdeletion size.

Read count was set to 10M in each sample. Detections 2Mb away from critical region are NOT reported.

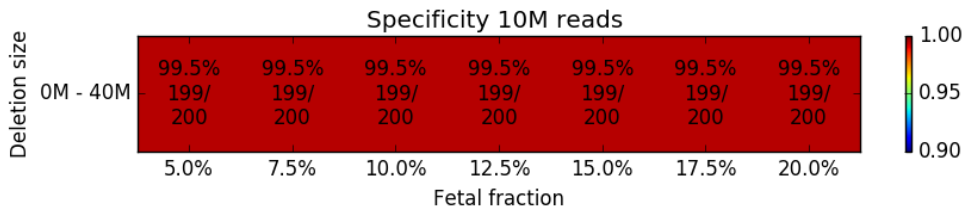

Fig H. Specificity of the prediction for different fetal fraction and microdeletion size.

Read count was set to 10M in each sample. Detections 2Mb away from critical region are NOT reported.

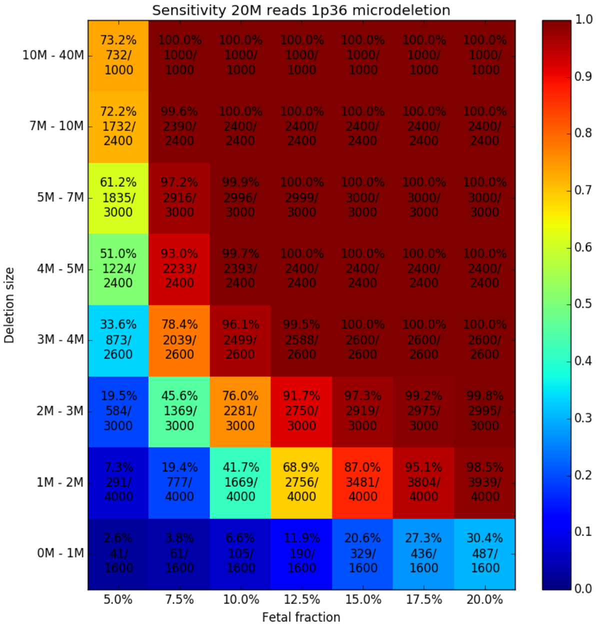
Sensitivity 20M reads 1p36 microdeletion
Deletion size
Fetal fraction
10M - 40M
73.2% 732/ 1000
100.0% 1000/ 1000
100.0% 1000/ 1000
100.0% 1000/ 1000
100.0% 1000/ 1000
100.0% 1000/ 1000
100.0% 1000/ 1000
7M - 10M
72.2% 1732/ 2400
99.6% 2390/ 2400
100.0% 2400/ 2400
100.0% 2400/ 2400
100.0% 2400/ 2400
100.0% 2400/ 2400
100.0% 2400/ 2400
5M - 7M
61.2% 1835/ 3000
97.2% 2916/ 3000
99.9% 2996/ 3000
100.0% 2999/ 3000
100.0% 3000/ 3000
100.0% 3000/ 3000
100.0% 3000/ 3000
4M - 5M
51.0% 1224/ 2400
93.0% 2233/ 2400
99.7% 2393/ 2400
100.0% 2400/ 2400
100.0% 2400/ 2400
100.0% 2400/ 2400
100.0% 2400/ 2400
3M - 4M
33.6% 873/ 2600
78.4% 2039/ 2600
96.1% 2499/ 2600
99.5% 2583/ 2600
100.0% 2600/ 2600
100.0% 2600/ 2600
100.0% 2600/ 2600
2M - 3M
19.5% 584/ 3000
45.6% 1369/ 3000
76.0% 2281/ 3000
91.7% 2750/ 3000
97.3% 2919/ 3000
99.2% 2975/ 3000
99.8% 2995/ 3000
1M - 2M
7.3% 291/ 4000
19.4% 777/ 4000
41.7% 1669/ 4000
68.9% 2756/ 4000
87.0% 3481/ 4000
95.1% 3804/ 4000
98.5% 3939/ 4000
0M - 1M
2.6% 41/ 1600
3.8% 61/ 1600
6.6% 105/ 1600
11.9% 190/ 1600
20.6% 329/ 1600
27.3% 436/ 1600
30.4% 487/ 1600
5.0% 7.5% 10.0% 12.5% 15.0% 17.5% 20.0%
0.0 0.1 0.2 0.3 0.4 0.5 0.6 0.7 0.8 0.9 1.0

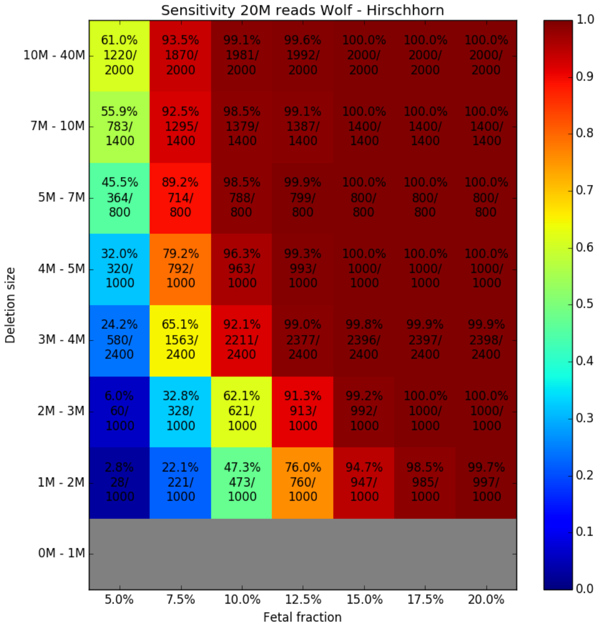
Sensitivity 20M reads Wolf - Hirschhorn
Deletion size
Fetal fraction
10M - 40M
61.0% 1220/ 2000
93.5% 1870/ 2000
99.1% 1981/ 2000
99.6% 1992/ 2000
100.0% 2000/ 2000
100.0% 2000/ 2000
100.0% 2000/ 2000
7M - 10M
55.9% 783/ 1400
92.5% 1295/ 1400
98.5% 1379/ 1400
99.1% 1387/ 1400
100.0% 1400/ 1400
100.0% 1400/ 1400
100.0% 1400/ 1400
5M - 7M
45.5% 364/ 800
89.2% 714/ 800
98.5% 788/ 800
99.9% 799/ 800
100.0% 800/ 800
100.0% 800/ 800
100.0% 800/ 800
4M - 5M
32.0% 320/ 1000
79.2% 792/ 1000
96.3% 963/ 1000
99.3% 993/ 1000
100.0% 1000/ 1000
100.0% 1000/ 1000
100.0% 1000/ 1000
3M - 4M
24.2% 580/ 2400
65.1% 1563/ 2400
92.1% 2211/ 2400
99.0% 2377/ 2400
99.8% 2396/ 2400
99.9% 2397/ 2400
99.9% 2398/ 2400
2M - 3M
6.0% 60/ 1000
32.8% 328/ 1000
62.1% 621/ 1000
91.3% 913/ 1000
99.2% 992/ 1000
100.0% 1000/ 1000
100.0% 1000/ 1000
1M - 2M
2.8% 28/ 1000
22.1% 221/ 1000
47.3% 473/ 1000
76.0% 760/ 1000
94.7% 947/ 1000
98.5% 985/ 1000
99.7% 997/ 1000
0M - 1M
5.0% 7.5% 10.0% 12.5% 15.0% 17.5% 20.0%
0.0 0.1 0.2 0.3 0.4 0.5 0.6 0.7 0.8 0.9 1.0

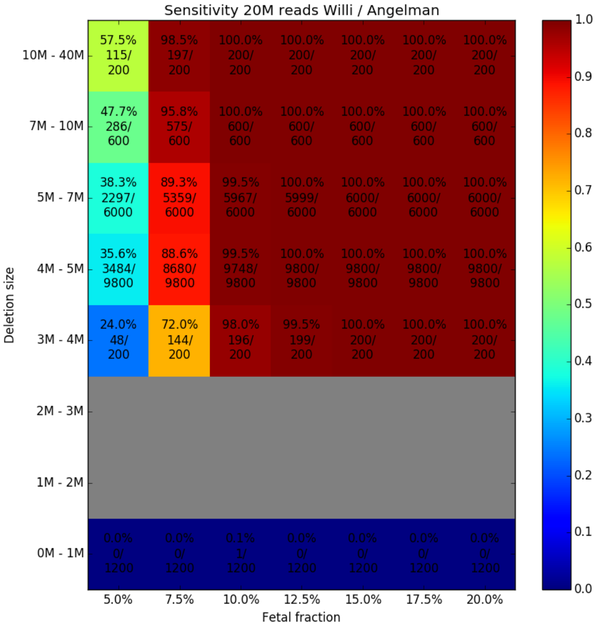
Sensitivity 20M reads Willi / Angelman
Deletion size
Fetal fraction
10M - 40M
57.5% 115/ 200
98.5% 197/ 200
100.0% 200/ 200
100.0% 200/ 200
100.0% 200/ 200
100.0% 200/ 200
100.0% 200/ 200
7M - 10M
47.7% 286/ 600
95.8% 575/ 600
100.0% 600/ 600
100.0% 600/ 600
100.0% 600/ 600
100.0% 600/ 600
100.0% 600/ 600
5M - 7M
38.3% 2297/ 6000
89.3% 5359/ 6000
99.5% 5967/ 6000
100.0% 5999/ 6000
100.0% 6000/ 6000
100.0% 6000/ 6000
100.0% 6000/ 6000
4M - 5M
35.6% 3484/ 9800
88.6% 8680/ 9800
99.5% 9748/ 9800
100.0% 9800/ 9800
100.0% 9800/ 9800
100.0% 9800/ 9800
100.0% 9800/ 9800
3M - 4M
24.0% 48/ 200
72.0% 144/ 200
98.0% 196/ 200
99.5% 199/ 200
100.0% 200/ 200
100.0% 200/ 200
100.0% 200/ 200
2M - 3M
1M - 2M
0M - 1M
0.0% 0/ 1200
0.0% 0/ 1200
0.1% 1/ 1200
0.0% 0/ 1200
0.0% 0/ 1200
0.0% 0/ 1200
0.0% 0/ 1200
5.0% 7.5% 10.0% 12.5% 15.0% 17.5% 20.0%
0.0 0.1 0.2 0.3 0.4 0.5 0.6 0.7 0.8 0.9 1.0

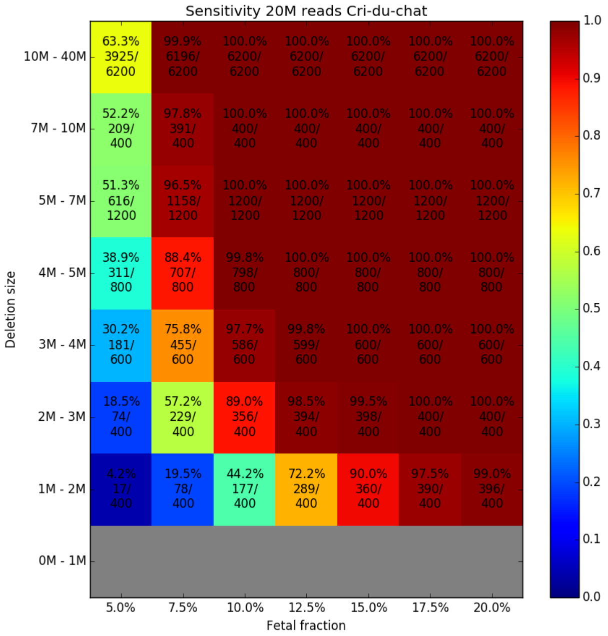
Sensitivity 20M reads Cri-du-chat
Deletion size
Fetal fraction
10M - 40M
63.3% 3925/ 6200
99.9% 6196/ 6200
100.0% 6200/ 6200
100.0% 6200/ 6200
100.0% 6200/ 6200
100.0% 6200/ 6200
100.0% 6200/ 6200
7M - 10M
52.2% 209/ 400
97.8% 391/ 400
100.0% 400/ 400
100.0% 400/ 400
100.0% 400/ 400
100.0% 400/ 400
100.0% 400/ 400
5M - 7M
51.3% 616/ 1200
96.5% 1158/ 1200
100.0% 1200/ 1200
100.0% 1200/ 1200
100.0% 1200/ 1200
100.0% 1200/ 1200
100.0% 1200/ 1200
4M - 5M
38.9% 311/ 800
88.4% 707/ 800
99.8% 798/ 800
100.0% 800/ 800
100.0% 800/ 800
100.0% 800/ 800
100.0% 800/ 800
3M - 4M
30.2% 181/ 600
75.8% 455/ 600
97.7% 586/ 600
99.8% 599/ 600
100.0% 600/ 600
100.0% 600/ 600
100.0% 600/ 600
2M - 3M
18.5% 74/ 400
57.2% 229/ 400
89.0% 356/ 400
98.5% 394/ 400
99.5% 398/ 400
100.0% 400/ 400
100.0% 400/ 400
1M - 2M
4.2% 17/ 400
19.5% 78/ 400
44.2% 177/ 400
72.2% 289/ 400
90.0% 360/ 400
97.5% 390/ 400
99.0% 396/ 400
0M - 1M
5.0% 7.5% 10.0% 12.5% 15.0% 17.5% 20.0%
0.0 0.1 0.2 0.3 0.4 0.5 0.6 0.7 0.8 0.9 1.0

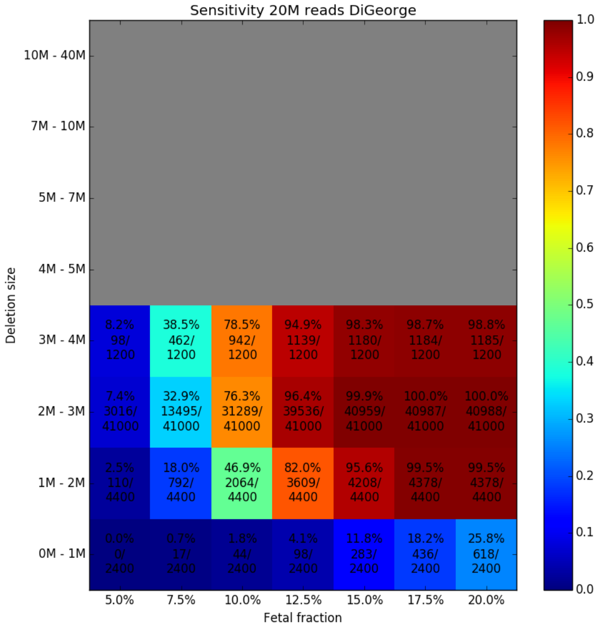

Fig I. Sensitivity of the prediction for different fetal fraction and size of microdeletion for different syndromes.

Read count was set to 20M in each sample. Detections 2M away from critical region are NOT reported.

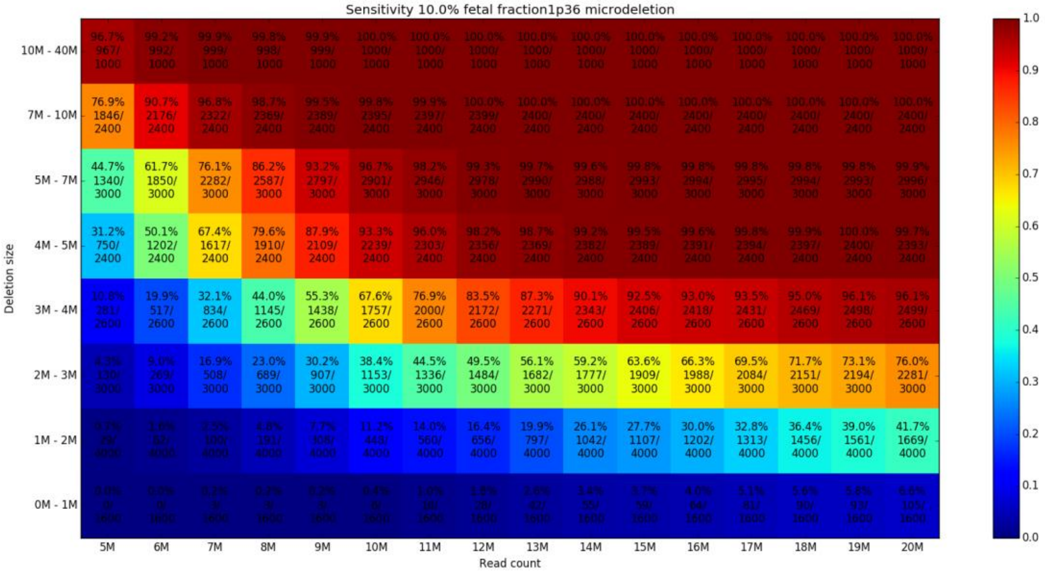
Sensitivity 10.0% fetal fraction1p36 microdeletion
Deletion size
10M - 40M
7M - 10M
5M - 7M
4M - 5M
3M - 4M
2M - 3M
1M - 2M
0M - 1M
5M 6M 7M 8M 9M 10M 11M 12M 13M 14M 15M 16M 17M 18M 19M 20M
Read count
1.0 0.9 0.8 0.7 0.6 0.5 0.4 0.3 0.2 0.1 0.0

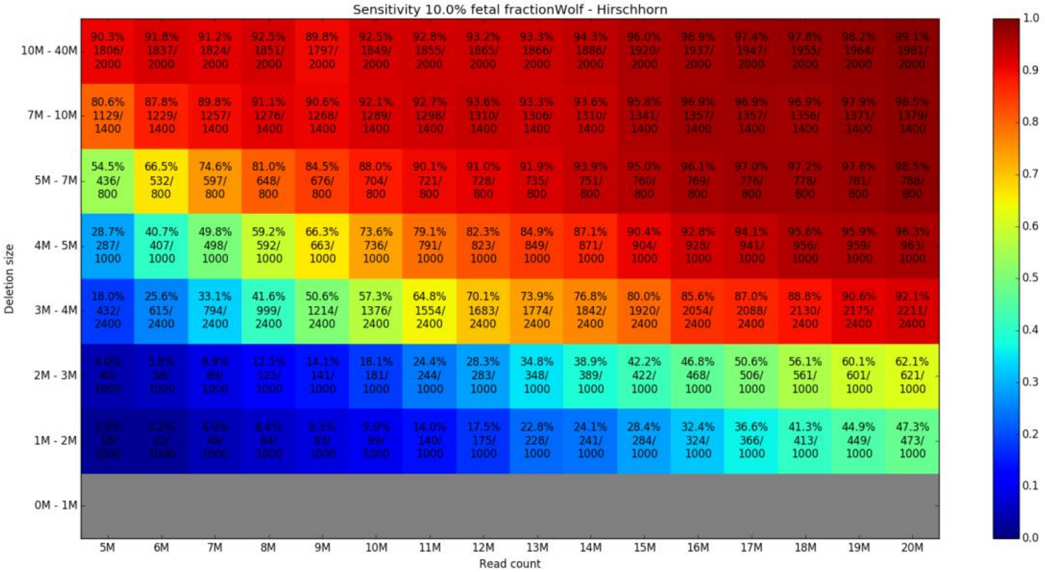
Sensitivity 10.0% fetal fractionWolf - Hirschhorn
Deletion size
10M - 40M
7M - 10M
5M - 7M
4M - 5M
3M - 4M
2M - 3M
1M - 2M
0M - 1M
5M 6M 7M 8M 9M 10M 11M 12M 13M 14M 15M 16M 17M 18M 19M 20M
Read count
1.0 0.9 0.8 0.7 0.6 0.5 0.4 0.3 0.2 0.1 0.0

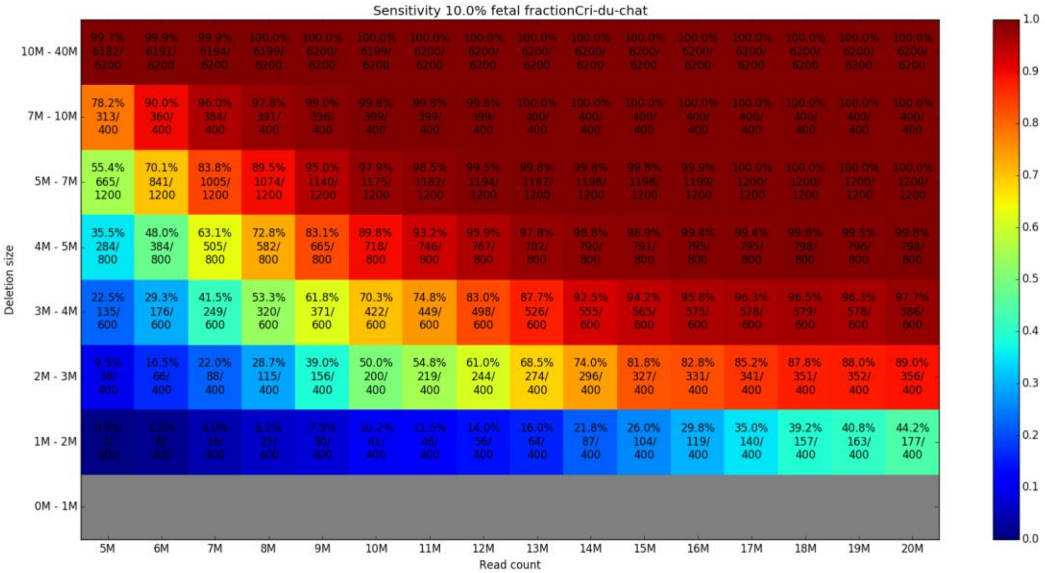
Sensitivity 10.0% fetal fractionCri-du-chat
Deletion size
10M - 40M
7M - 10M
5M - 7M
4M - 5M
3M - 4M
2M - 3M
1M - 2M
0M - 1M
5M 6M 7M 8M 9M 10M 11M 12M 13M 14M 15M 16M 17M 18M 19M 20M
Read count
1.0 0.9 0.8 0.7 0.6 0.5 0.4 0.3 0.2 0.1 0.0

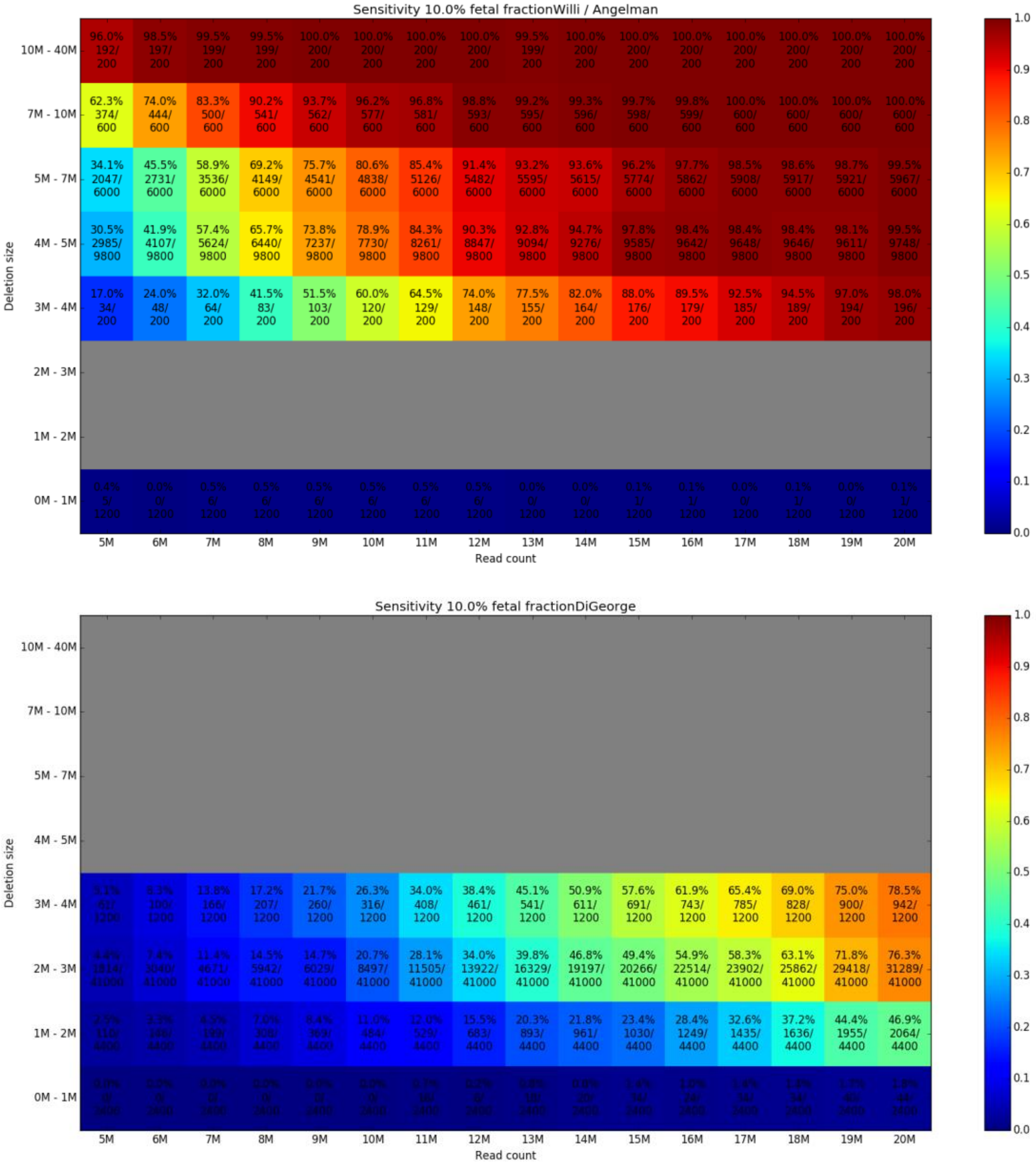

Fig J. Sensitivity of the prediction for different read count and microdeletion size.

Fetal fraction was set to 10% for all samples. Detections 2M away from critical region are NOT reported.

Table A. Detailed description of the control samples and their detection accuracy.

name	read count	fetal fraction	detection	local z score	size	syndrome
NA17942	17.5M	4.15%	ND	ND	2.6M	DiGeorge
BA161216	25.27M	4.50%	ND/grey zone	-2,48	3M	DiGeorge
NA22936	15.9M	4.86%	D	-5,28	21M	1p36 microdeletion
NA14124	16.47M	5.11%	D	-8,35	17.7M	Cri-du-chat
BA34	19.24M	5.90%	ND	ND	0.9M	DiGeorge
NA09024	15.3M	7.30%	D	-4,82	5.3M	Willi / Angelman
NA09024	12.66M	7.60%	D	-5,79	5.3M	Willi / Angelman
NA17942	19.6M	8.70%	D	-4,29	2.6M	DiGeorge
BA34	11M	8.80%	ND	ND	0.9M	DiGeorge
NA00072	8.3M	9.10%	D	-16,98	9.3M	Wolf - Hirschhorn
NA22936	21.5M	9.85%	D	-10,66	21M	1p36 microdeletion
NA11515	19.31M	10.60%	D	-5,59	6M	Willi / Angelman
BA34	9.9M	10%	ND	ND	0.9M	DiGeorge
BA161216	20.59M	11.20%	D	-4,71	3M	DiGeorge
BA34	20.36M	11.50%	D	-5,34	0.9M	DiGeorge
BA34	8.9M	11.99%	D	-5,74	0.9M	DiGeorge
NA11515	16.79M	12.20%	D	-9,02	6M	Willi / Angelman
NA09024	24.3M	13.40%	D	-7,38	5.3M	Willi / Angelman
NA11515	15.71M	13.90%	D	-9,54	6M	Willi / Angelman
NA00072	8.4M	14.10%	D	-14,62	9.3M	Wolf - Hirschhorn
NA09024	15.37M	14.50%	D	-10.25/-5.58	5.3M	Willi / Angelman
NA11515	19.26M	14.60%	D	-7,72	6M	Willi / Angelman
NA00072	16.2M	16.40%	D	-14,3	9.3M	Wolf - Hirschhorn
NA17942	14.5M	16.69%	D	-7,11	2.6M	DiGeorge
BA34	21.52M	17.30%	D	-6,52	0.9M	DiGeorge
NA17942	19.45M	17.30%	D	-6,71	2.6M	DiGeorge
BA161216	20.39M	20.10%	D	-8,27	3M	DiGeorge
NA17942	20.39M	20.10%	D	-8,27	2.6M	DiGeorge
NA09024	16.1M	20.50%	D	-13.50/-5.71	5.3M	Willi / Angelman

Table B. The number, mean size of pathogenic regions, and resulting critical regions, as reported by our study and DECIPHER database.

The number of pathogenic regions overlapping its corresponding critical region is reported in brackets. Finally, the number of pathogenic deletions from DECIPHER database that does not overlap ISCA database is low.

Identification of a syndrome		Number of pathogenic deletions		Mean size of pathogenic deletions	
**Band**	**Name**	**ISCA**	**DECIPHER**	**ISCA**	**DECIPHER**
1p36	1p36 deletion	102 (100)	54 (52)	4,131,271	3,174,263
4p16.3	Wolf-Hirschhorn	48 (48)	27 (27)	6,713,778	4,819,991
5p15	Cri-du-chat	50 (50)	24 (24)	14,887,247	8,873,724
15q11	Angelman / Prader-Willi	90 (90)	133 (133)	5,061,279	2,534,265
22q11.21	DiGeorge	280 (245)	136 (120)	2,368,692	2,444,038

Critical regions		Pathogenic deletions from DECIPHER not in ISCA critical region
**ISCA**	**DECIPHER**	
chr1: 564,424 - 21,598,492	chr1: 120,840 - 22,542,549	3
chr4: 85,040 - 2,010,761	chr4: 71,552 - 5,506,588	2
chr5: 1 - 15,678,560	chr5: 113,576 - 18,992,827	2
chr15: 22,779,922 - 28,559,437	chr15: 22,373,311 - 28,560,803	2
chr22: 18,661,724 - 21,505,417	chr22: 18,890,162 - 21,464,119	15
